# Integrated analysis of coding genes and non-coding RNAs during hair follicle cycle of cashmere goat (*Capra hircus*)

**DOI:** 10.1186/s12864-017-4145-0

**Published:** 2017-10-11

**Authors:** Shanhe Wang, Wei Ge, Zhixin Luo, Yang Guo, Beilei Jiao, Lei Qu, Zhiying Zhang, Xin Wang

**Affiliations:** 1 College of Animal Science & Technology, Northwest A&F University, Yangling, Shaanxi 712100 China; 2 Life Science Research Center, Yulin University, Yulin, Shaanxi 719000 China

**Keywords:** Cashmere goat, Anagen, Telogen, Hair follicle, lncRNA, miRNA

## Abstract

**Background:**

Cashmere growth is a seasonal and cyclic phenomenon under the control of photoperiod and multiple stimulatory and inhibitory signals. Beyond relevant coding genes, microRNA (miRNA) and long non coding RNA (lncRNA) play an indispensable role in hair follicle (HF) development and skin homeostasis. Furthermore, the influence of lncRNA upon miRNA function is also rapidly emerging. However, little is known about miRNAs, lncRNAs and their functions as well as their interactions on cashmere development and cycling.

**Result:**

Here, based on lncRNA and miRNA high-throughput sequencing and bioinformatics analysis, we have identified 1108 lncRNAs and 541 miRNAs in cashmere goat skin during anagen and telogen. Compared with telogen, 1388 coding genes, 41 lncRNAs and 15 miRNAs were upregulated, while 1104 coding genes, 157 lncRNAs and 8 miRNAs were downregulated in anagen (adjusted *P*-value ≤0.05 and relative fold-change ≥2). Subsequently, we investigated the impact of lncRNAs on their target genes in cis and trans, indicating that these lncRNAs are functionally conserved during HF development and cycling. Furthermore, miRNA-mRNA and miRNA-lncRNA interaction were identified through the bioinformatics algorithm miRanda, then the ceRNA networks, miR-221-5p-lnc_000679-*WNT3*, miR-34a-lnc_000181-*GATA3* and miR-214-3p-lnc_000344-*SMAD3*, were constructed under defined rules, to illustrate their roles in cashmere goat HF biology.

**Conclusion:**

The present study provides a resource for lncRNA, miRNA and mRNA studies in cashmere cycling and development. We also demonstrate potential ceRNA regulatory networks in cashmere goat HF cycling for the first time. It expands our knowledge about lncRNA and miRNA biology as well as contributes to the annotation of the goat genome.

**Electronic supplementary material:**

The online version of this article (10.1186/s12864-017-4145-0) contains supplementary material, which is available to authorized users.

## Background

*Capra hircus*, an economically important livestock species, plays an indispensable role in the world animal fiber industry [1]. Shanbei white cashmere goat, a double-coated (wool and cashmere) species famous for its high and luxurious fiber production traits, exhibits seasonal rhythms with a well-defined duration of fiber growth and have been an excellent cashmere goat breed in China.

Cashmere growth is a seasonal phenomenon under the control of photoperiod and the animals’ endocrine systems [2–4]. The cashmere of the Shanbei white cashmere goat undergoes cyclic transformation from the resting phase (telogen, May to July, summer) to the growth phase (anagen, July to February). The latter phase is characterized by rapid proliferation of follicular keratinocytes and elongation and thickening of the hair shaft. The regression phase (catagen, March to May, spring) leads to the degeneration of the HF. The basis for HF cycle rests in the unique follicular epithelial and mesenchymal components and their interactions [5, 6]. Recently, some molecular signals such as fibroblast growth factor, transforming growth factor-β, WNT signaling pathway, sonic hedgehog, neurotrophins, and homeobox [7–11], and their interactions have been defined [12–14]. These gene families were also found in other regenerating systems [15, 16].

The recent explosion in knowledge demonstrating the importance of miRNA and lncRNA in the regulation of multiple major biological processes impacting development, differentiation, and metabolism have brought these heretofore neglected molecular players to the forefront [17–19]. Specifically, for the biology of HF, the miRNA-processing enzyme Dicer was essential for the morphogenesis and maintenance of HF [20]. Meanwhile, another miRNA-processing enzyme Drosha was required for HF regression, hair shaft differentiation, long-term maintenance of HF stem cells, and epidermal homeostasis [21]. Since then more miRNAs have been identified and characterized in HF biology, such as miR-214, miR-21 and miR-24 [22–24]. Researchers have identified several functional lncRNAs associated with skin biology, such as ANCR, TINCR, U1 RNA, PRINS, BANCR, and SPRY4-IT1 [25]. Furthermore, lncRNA in dermal papilla cells contributes to regulating the genes involved in hair follicle development and postnatal hair cycling [26]. Even more important, the influence of lncRNAs on microRNA function is also rapidly emerging [27–29], the microRNA-lncRNA regulatory paradigms modulate gene expression patterns that drive major cellular processes (such as cell differentiation, proliferation, and cell death) which are central to mammalian physiologic and pathologic processes [30, 31].

However, previous findings mainly focused on human or mouse, little was known about miRNAs, lncRNAs and their functions on cashmere development and cycling [19, 32]. In this current study, the coding genes, lncRNA as well as miRNA profile of cashmere goat skin in anagen and telogen were detected using deep-sequencing. Subsequently, the function of key genes, miRNAs, lncRNAs and their potential interactions on cashmere development and cycling were analyzed using bioinformatics. This study will expand our knowledge about lncRNA and miRNA in HF biology as well as contribute to the annotation of the goat genome.

## Methods

### Animals and samples

Shanbei cashmere goats with the fine fiber production trait were used in this study. All the goats were obtained from Shanbei cashmere goats engineering technology research center of Shaanxi province, China. The experimental animals were fed according to the local cashmere goat standard of Shaanxi (DB61/T583-2013, http://www.sxny.gov.cn/). Six female adults (1 year old, coefficient of relationship <0.125) were selected. After intravenous injection of lidocaine hydrochloride, skin samples approximately 2 cm^2^ and 3 mm deep were harvested from the body side of adult goats at distinct hair cycle stages (anagen and telogen), frozen in sample protector for RNA (Takara, China) and stored at −80 °C for future analysis. The same animals were collected at an adjacent site at both anagen and telogen.

All the experimental procedures with goats used in the present study had been given prior approval by the Experimental Animal Manage Committee of Northwest A&F University (2011-31101684).

### RNA isolation, library preparation, and sequencing

Total RNA was extracted from the collected skin tissues using Trizol reagent (Invitrogen, USA) following the manufacturer’s instructions, after grinding them in liquid nitrogen. The RNA concentration and quality was determined using the Agilent 2100 Bioanalyzer. The extracted total RNA was stored at −80 °C for later use.

For lncRNA sequencing, a total amount of 3 μg RNA per sample was used as input material for the RNA library preparations. Firstly, ribosomal RNA was removed using the Epicentre Ribo-zero™ rRNA Removal Kit (Epicentre, USA), and the rRNA was cleaned up by ethanol precipitation. Subsequently, in total six libraries from anagen (*n* = 3) and telogen (*n* = 3) were generated from the rRNA-depleted RNA using the NEBNext® Ultra™ Directional RNA Library Prep Kit for Illumina® (NEB, USA) following the manufacturer’s recommendations. Strand-specific sequencing was performed on the Illumina Hiseq 4000, PE 150 system for these libraries (paired-end 100-bp reads).

For miRNA sequencing, the six animals at each stage were randomly divided into two groups, and the RNA from three single goats per group was pooled. Four RNA pool libraries from telogen (*n* = 2) and anagen (*n* = 2) were constructed. Small RNA fragments of 18-30 nt in length were isolated and purified from total RNA using 15% denaturing polyacrylamide gel electrophoresis (PAGE) using a gel extraction kit (Sangon Biotech, China). Subsequently, 3′ and 5′ RNA adaptors were ligated to the RNA pool using NEBNext® Multiplex Small RNA Library Prep Set for Illumina® (NEB, USA.) following the manufacturer’s recommendations, and index codes were added to attribute sequences to each sample. Then, first-strand cDNA was synthesized using M-MuLV Reverse Transcriptase (RNase H–) (NEB, USA.). PCR amplification was performed using LongAmp Taq 2× Master Mix (NEB, USA.), SR Primer for illumina and index (X) primer for 16 cycles. PCR products were purified on a 10% polyacrylamide gel (100 V, 80 min). DNA fragments corresponding to 140~160 bp (the length of small noncoding RNA plus the 3′ and 5′ adaptors) were recovered and rehydrated in 8 μL elution buffer (OMEGA bio tek, USA.). Then, library quality was assessed on the Agilent Bioanalyzer 2100 system using DNA High Sensitivity Chips. The four library preparations were sequenced on an Illumina Hiseq 2500 platform and 50 bp single-end reads were generated.

### Quality control

Raw data were first processed using in-house Perl scripts. In this step, clean data were obtained by trimming reads containing adapter, reads containing over 10% of ploy-N, and low-quality reads (>50% of bases whose Phred scores were <5) from the raw data. The Phred score [33] (Q20, Q30) and GC content of the clean data were calculated. All subsequent analysis was based on the high-quality data.

### Transcriptome assembly

The high quality reads were mapped independently to the goat genome v2.0 (ftp://ftp.ncbi.nlm.nih.gov/genomes/all/GCA/000/317/765/GCA_000317765.2_CHIR_2.0) using Bowtie v2.0.6 [34] and the spliced read aligner TOPHAT v2.0.9 (main parameter: library-type <fr- firststrand>) [35]. The mapped reads of each sample were assembled using Cufflinks (v2.1.1) in a reference-based approach [36]. Cufflinks was run with‘min-frags-per-transfrag = 0’ and ‘--library-type’, other parameters were set as default. We then adopted five steps to identify goat lncRNAs from the assembled transcripts: (1) transcripts with length < 200 bp were removed; (2) transcripts with exon number < 2 were removed; (3) transcripts were compared with mRNA, rRNA, tRNA, snRNA, snoRNA and pre-miRNA (https://www.ncbi.nlm.nih.gov/) using Cuffcompare v2.1.1 to remove the same or similar transcripts [36]. (4) transcripts with FPKM <0.5 were removed; (5) transcripts that did not pass the protein-coding-score test were removed using the Coding Potential Calculator (CPC) [37], PFAM database [38] and Coding-Non-Coding Index (CNCI) software [39]. CNCI was used with default parameters. For CPC, the NCBI eukaryotes’ protein database was used and the e-value was set to ‘1e-10′. For Pfam-scan, each transcript was translated in all three possible frames and Pfam Scan (v1.3) was used to identify the occurrence of any of the known protein family domains documented in the Pfam database (release 27; used both Pfam A and Pfam B) (http://pfam.xfam.org/). Any transcript with a Pfam hit was excluded from the following steps. Pfam searches used default parameters of -E 0.001 --domE 0.001.

### Known miRNA alignment and novel miRNA prediction

Mapped small RNA tags were used to look for known miRNA. MiRBase20.0 (http://www.mirbase.org/) [40] was used as a reference, modified software mirdeep2 [41] and srna-tools-cli were used to obtain the potential miRNA and draw the secondary structures. Custom scripts were used to obtain the miRNA counts, as well as base bias at the first position of identified miRNA with certain length, and on each position of all identified miRNA respectively. To remove tags originating from protein-coding genes, repeat sequences, rRNA, tRNA, snRNA, and snoRNA, small RNA tags were mapped to RepeatMasker (http://www.repeatmasker.org/), Rfam 12.0 database (http://rfam.xfam.org/) [42] or those types of data (https://www.ncbi.nlm.nih.gov/) from goat.

The characteristic hairpin structure of miRNA precursors can be used to predict novel miRNA. The available software miREvo [43] and mirdeep2 [41] were integrated to predict novel miRNA through exploring the secondary structure, the Dicer cleavage site and the minimum free energy of small RNA tags unannotated in the former steps. At the same time, custom scripts were used to obtain the identified miRNA counts as well as base bias on the first position with certain length and on each position of all identified miRNA respectively.

### Quantification of gene expression level

Cuffdiff (v2.1.1) [36] was used to calculate fragments per kb per million reads (FPKM) of both lncRNAs and coding genes in each sample. It was also used to provide statistical routines for determining differential expression in gene expression data using a model based on the negative binomial distribution. Transcripts or genes with a *P*-adjust ≤0.05 [44] and fold change ≥2 were described as differentially expressed between anagen and telogen. MiRNA expression levels were estimated by TPM (transcript per million) with the following criteria [45]: Normalization formula: Normalized expression = actual miRNA count/total count of clean reads*1000000; Differential expression analysis of two groups was performed using the DESeq R package (1.8.3). The *P*-values were adjusted using the Benjamini and Hochberg method [44]. A corrected *P*-value of 0.05 was set as the threshold for significantly differential expression by default. Scatter plots were used to demonstrate differentially expressed miRNA between the two follicular stages.

### LncRNA target gene prediction

To explore the function of lncRNAs, we first predicted the target genes of lncRNAs in cis and trans. The cis role refers to lncRNAs’ action on neighboring target genes. In the present study, the coding genes from 100 kb upstream and downstream of an lncRNA were searched. The trans role refers to the influence of lncRNAs on other genes at the expression level. Pearson’s correlation coefficients were calculated between expression levels of lncRNAs and mRNAs with custom scripts (Pearson correlation ≥0.95 or ≤ −0.95).

### Validation of gene expression in RNA-seq by quantitative PCR analysis

The total RNAs from the goats used for RNA-seq were also used for quantitative PCR analysis. For lncRNAs and mRNAs, the first-strand cDNA was obtained using a PrimeScript™ RT reagent Kit with gDNA Eraser (TAKARA, China), and then were subjected to quantification of the mRNAs and lncRNAs with β-actin as an endogenous control using SYBR® *Premix Ex Taq*™ II (TAKARA, China) on the Bio-Rad CFX96 Touch™ Real Time PCR Detection System. The quantitative PCR was performed using the following conditions: 95 °C for 60 s, 40 cycles of 95 °C for 10 s, and the optimized annealing temperature for 30 s. The primers and annealing temperatures for genes are listed in Additional file 1.

For miRNA quantitative PCR, the first-strand cDNA was obtained using Mir-X™ miRNA First Strand Synthesis Kit (Clontech, China), and then were subjected to quantification of miRNAs with U6 as an endogenous control as described above. The forward primer of specific miRNA was the sequence of the mature miRNA itself, the reverse was universal primer, which was provided in the kit. The primers for U6 were also provided in the kit. The quantitative PCR was performed on an CFX96 Touch™ (Bio-Rad, USA.) using the following conditions: 95 °C for 10 s, 40 cycles of 95 °C for 5 s, and the optimized annealing temperature for 20 s.

Each stage (anagen and telogen) included at least 3 samples, and all reactions were performed in triplicate for each sample. Gene expression was quantified relative to endogenous gene expression using the comparative cycle threshold (ΔCT) method [46] through Bio-Rad CFX Manager 3.1 and Microsoft excel 2013. Differences in gene expression between the anagen and telogen were detected by independent sample *t*-test.

### Bioinformatics analysis

Predicted interactions between miRNA and lncRNA were determined using miRanda. Predicted targets of differentially expressed miRNAs were determined using miRanda and targetscan (http://www.targetscan.org/ and http://www.microrna.org/microrna/). In addition, Pathway analysis was used to identify significant pathways for the differentially expressed genes according to the Kyoto Encyclopedia of Genes and Genomes (KEGG) (http://www.genome.jp/kegg/) [47]. We used KOBAS software (main parameter: blastx 1e-10; padjust: BH) [48] to test the statistical enrichment of differentially expressed genes or lncRNA target genes in KEGG pathways.

### CeRNA network construction

We predicted lncRNAs that might act as ceRNAs according to the following steps. (i) lncRNA screening: lncRNAs that were up- or down-regulated fold change >2.0 and *P*-adjust value <0.05 were first retained; (ii) miRNAs associated with HF development and cycling were retained, including miR-148b, miR-183, miR-184, miR-196a, miR-199a, miR-21, miR-24, miR-200, miR-200b, miR-203, miR-205, miR-214-3p, miR-221, miR-222, miR-31, miR-34a and miR-34c; (iii) lncRNA-mRNA interaction were obtained based on expression correlation coefficient (Pearson correlation ≥0.95), the DEGs were retained; (iv) lncRNA-miRNA interactions were predicted by miRanda; (v) mRNAs targeted by miRNAs were predicted by miRanda and targetscan. The software Cytoscape 3.4.0 was used to graphically visualize networks [49].

## Results

### Identification of lncRNAs in cashmere goat skin

**Fig. 1:**
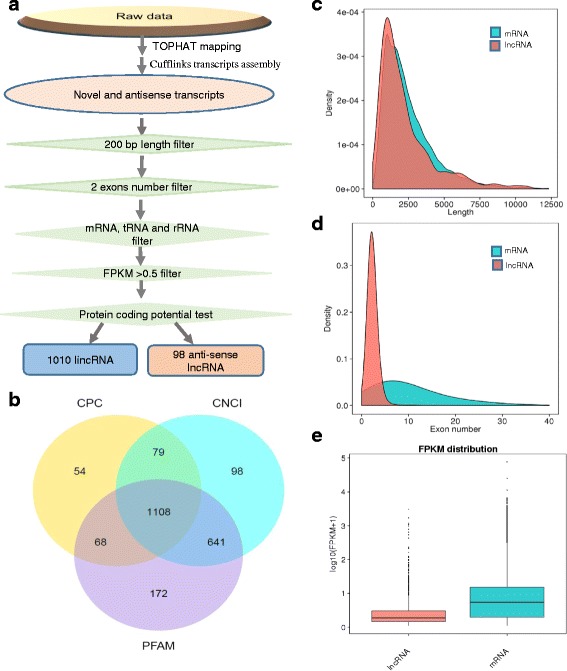
Identification and characterization of long noncoding RNAs (lncRNAs) in *Capra hircus*. **a** The pipeline of lncRNA identification in *Capra hircus*. **b** Screening of the candidate lncRNAs in skin transcriptome. Venn diagrams of coding potential analysis by using stringent criteria. Three tools (CPC, CNCI and PFAM) were employed to analyze the coding potential of lncRNAs. Those simultaneously shared by three analytical tools were designated as candidate lncRNAs and used in subsequent analysis. **c** Distribution of transcript lengths in the lncRNAs and protein-coding transcripts. **d** Distribution of exon number in the lncRNAs and protein-coding transcripts. **e** Expression level indicated by log_10_ (FPKM + 1) in the mRNAs and lncRNAs

In order to develop a comprehensive catalogue of lncRNAs in goat, a prerequisite was to integrate the high-quality and high-depth RNA-seq data set. To determine the orientation of transcripts accurately, we generated six transcriptomes covering different cycling stages of goat cashmere using the stranded sequencing method. A total of 644,884,566 raw reads were produced from the Illumina PE 150 platform. After trimming to remove adaptor sequences, and discarding low-quality sequences, we retained 623,168,524 clean reads (93.5 Gb, accounting for 96.63% of raw reads). Subsequently, we mapped the clean reads to the latest goat reference genome v2.0 (https://www.ncbi.nlm.nih.gov/assembly/GCA_000317765.2) using the TOPHAT–CUFFLINKS pipeline [36]. Some filtering steps were conducted to retain bona fide lncRNAs (Fig. 1a). After coding potential analysis using the software CNCI, CPC and Pfam-scan, 1108 lncRNA loci including 1010 long intergenic non-coding RNA (lincRNAs) and 98 anti-sense lncRNAs were identified (Fig. 1b) (Additional file 2). The sequence information of lncRNAs is shown in Additional file 3.

### Comparison of features of mRNAs and lncRNAs

To comprehensively examine the differences between lncRNAs and the protein coding transcripts, comparative analysis was performed on gene structure, expression, and sequence conservation. The results showed that goat lncRNAs were slightly shorter than mRNAs in length distribution (Fig. 1c). Moreover, the number of exons and expression level were also less than that of mRNAs (Fig. 1d & e).

### Identification of miRNAs in cashmere goat skin

In order to identify miRNAs involved in the cashmere fiber cycle, four small RNA (sRNA) libraries representing anagen and telogen were constructed. Each library was a mixed pool from three adult cashmere goat skin samples. A total of 54,892,902 raw reads were obtained. After discarding the sequences shorter than 18 nt, eliminating low-quality sequences and removing contaminants formed by adapter–adapter ligation, reads without 3′ ligation and insert tags were obtained. Collectively, a total of 54,013,578 clean reads were retained for further analysis. Among these sequences, most were distributed in the 18-24 nt range. The highest percentage of these sRNAs were 22 nt long, which is consistent with the common size of miRNAs. All the clean reads (54,013,578) were aligned with the goat genome sequence using bowite v2.0.6 software [34]. Among them, 42,018,732 (77.79%) clean reads were mapped in the goat genome. As a result, 411 annotated mature miRNAs from 259 precursors were identified (Additional file 4), which represented 10.86% of the total reads and 0.22% of the unique clean reads. Additionally, 130 novel mature miRNAs and 139 miRNA precursors were found using miREvo [43] and mirdeep2 (Additional file 5).

### Differentially expressed genes (DEGs) and non-coding RNAs (ncRNAs, miRNA and lncRNA)

**Fig. 2:**
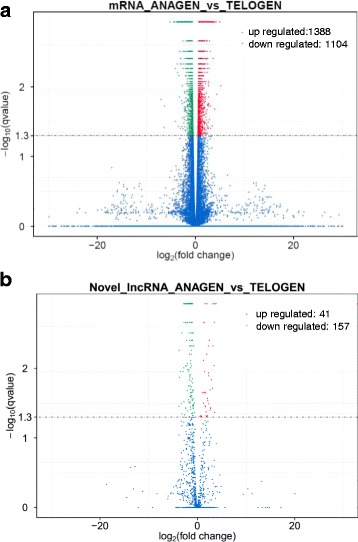
Differentially expressed coding genes and lncRNAs in goat skin between anagen and telogen of HF cycle (Fold Change ≥2 and *P*-adjust value ≤0.05). **a** Differentially expressed coding genes. **b** Differentially expressed lncRNAs. Among these genes and lncRNAs, 1385 coding genes and 37 lncRNAs were upregulated, and 1104 coding genes and 156 lncRNAs were downregulated in anagen compared with telogen. Green dot indicates coding gene or lncRNA down regulated, red dot indicates coding gene or lncRNA up regulated in anagen compared with telogen

The mRNA and lncRNA expression levels were analyzed using Cuffdiff v2.1.1 [36]. The overall expression levels of both lincRNAs and antisense-lncRNA were lower than that of protein-coding transcripts, which was consistent with a previous study [19]. Using edgeR (the threshold is usually set as Fold Change ≥2 and *P*-adjust value ≤0.05), the differentially expressed lncRNAs and genes between anagen and telogen were screened, resulting in 2492 DEGs (Additional file 6) and 198 differentially expressed lncRNAs (Additional file 7). Among these genes and lncRNAs, 1388 genes and 41 lncRNAs were upregulated, and 1104 genes and 157 lncRNAs were downregulated in anagen compared with telogen (Fig. 2).

It was noteworthy that our analysis identified a set of genes belonging to keratin family member encoding genes (KRT) and keratin-associated protein (KAP), which were markedly up-regulated in anagen compared with telogen (Additional file 8). Previous studies have shown that KRT and KAP are major structural proteins of hair fiber and sheath, and their contents were important for fleece quality [50].

Meanwhile, a few lncRNAs were specifically expressed at a single developmental stage of cashmere cycling, such as Lnc_00092, Lnc_000183, Lnc_000406 and Lnc_000559, which showed telogen-specific expression, while Lnc_000173 showed anagen-specific expression, indicating that these lncRNAs could regulate cashmere cycling through their spatio-temporal expression.

As to miRNAs analysis, 21 known miRNAs and 2 novel miRNAs were found to have significantly different expression between anagen and telogen. Among them, 8 miRNAs were down-regulated and 15 miRNAs were up-regulated compared with telogen (Additional file 9). Among these miRNAs, miR-214-3p, miR-196a and miR-34c-5p had been reported with HF development and cycling functions [20, 24, 32].

**Fig. 3:**
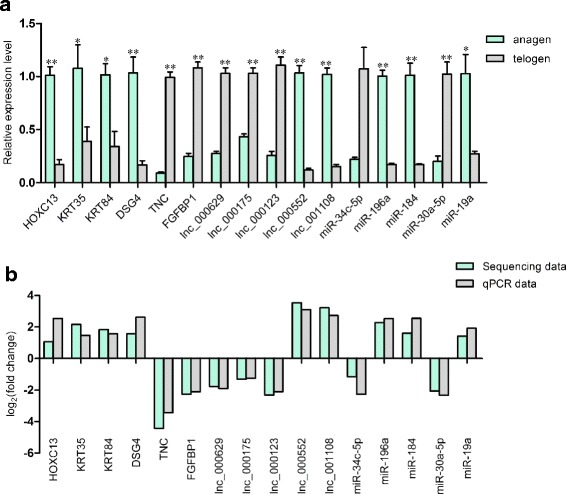
Sequencing data validated by qPCR. **a** The expression level of differently expressed genes, miRNAs and lncRNAs validated by qPCR. Coding gene and lncRNA expression was quantified relative to the expression level of β-actin, miRNA expression was quantified relative to the expression level of U6 using the comparative cycle threshold (ΔCT) method. The data are expressed as the mean ± 1 SE (*n* = 3). * *P* < 0.05, **P < 0.01 (**b**) Comparison the expression pattern of the sequencing data and qPCR data. Log_2_ (fold change) > 0 indicates the transcript up regulated in anagen compared to telogen. Log_2_ (fold change) < 0 indicates the transcript down regulated in anagen compared to telogen

To confirm the expression patterns of the genes, we randomly selected 5 lncRNAs, 6 mRNAs and 5 miRNAs and validated their expression patterns using quantitative real-time PCR (qRT-PCR). The results were in concordance with the RNA-seq data, suggesting that the expression patterns based on RNA-seq data were reliable (Fig. 3).

### KEGG analysis of DEGs

**Fig. 4:**
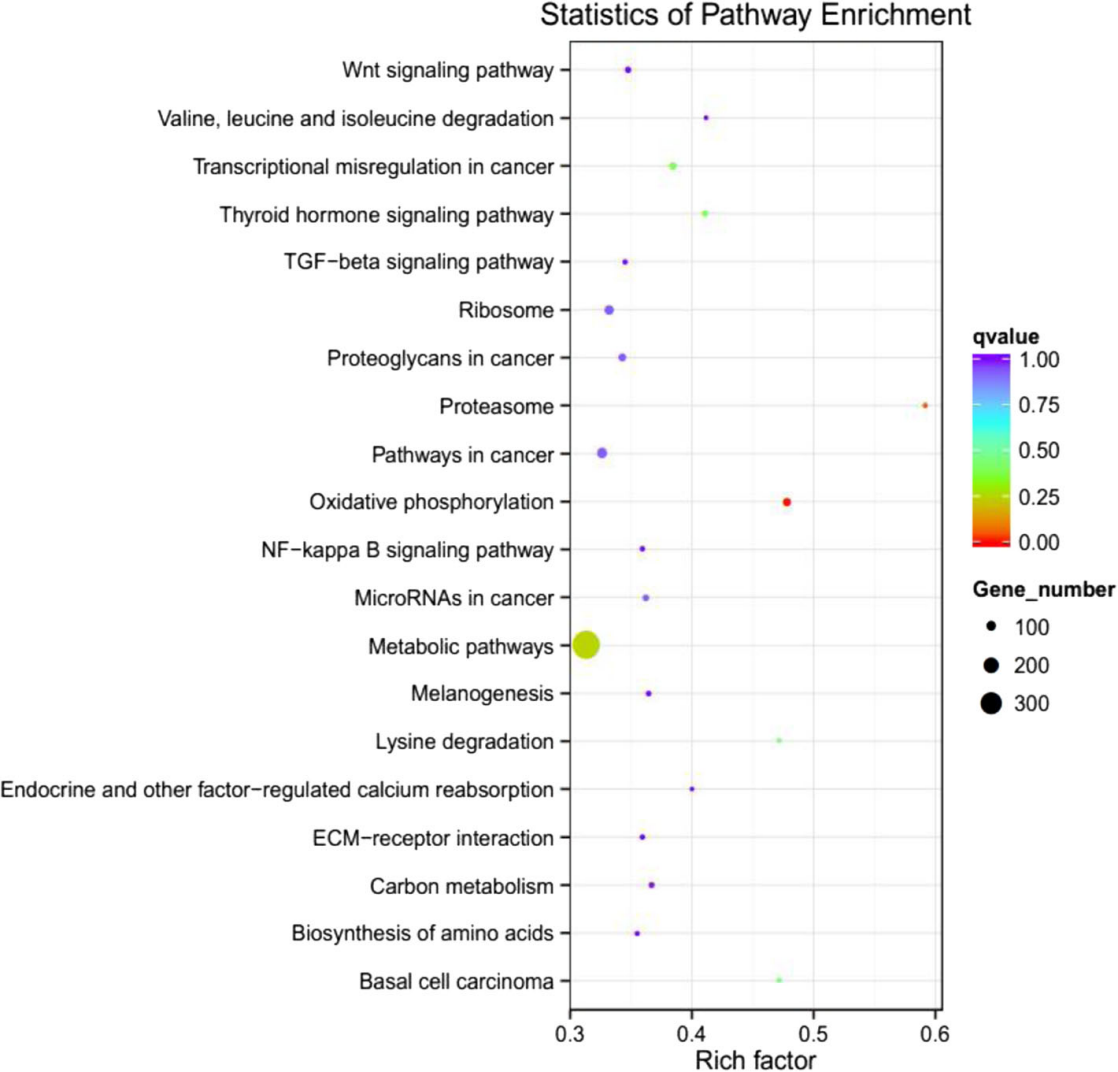
The top 20 KEGG pathways of differentially expressed genes in goat skin between anagen and telogen. Rich factor indicates the ratio of DEGS enriched in the pathway among genes annotated in the pathway

KEGG analysis predicted that the DEGs were enriched in 274 pathways. The top 20 KEGG pathways with the highest representation of DEGs are shown in Fig. 4. Among the identified KEGG pathways, some belonged to conventional pathways associated with HF cycling, such as the WNT signaling pathway, ECM-receptor interaction, TGF-β signaling pathway and VEGF signaling pathway. However, the oxidative phosphorylation pathway, proteasome pathway, metabolic pathway and lysine degradation pathways were up regulated, therefore may play an important role in HF cycling.

### The cis and trans role of lncRNAs in target genes

**Fig. 5:**
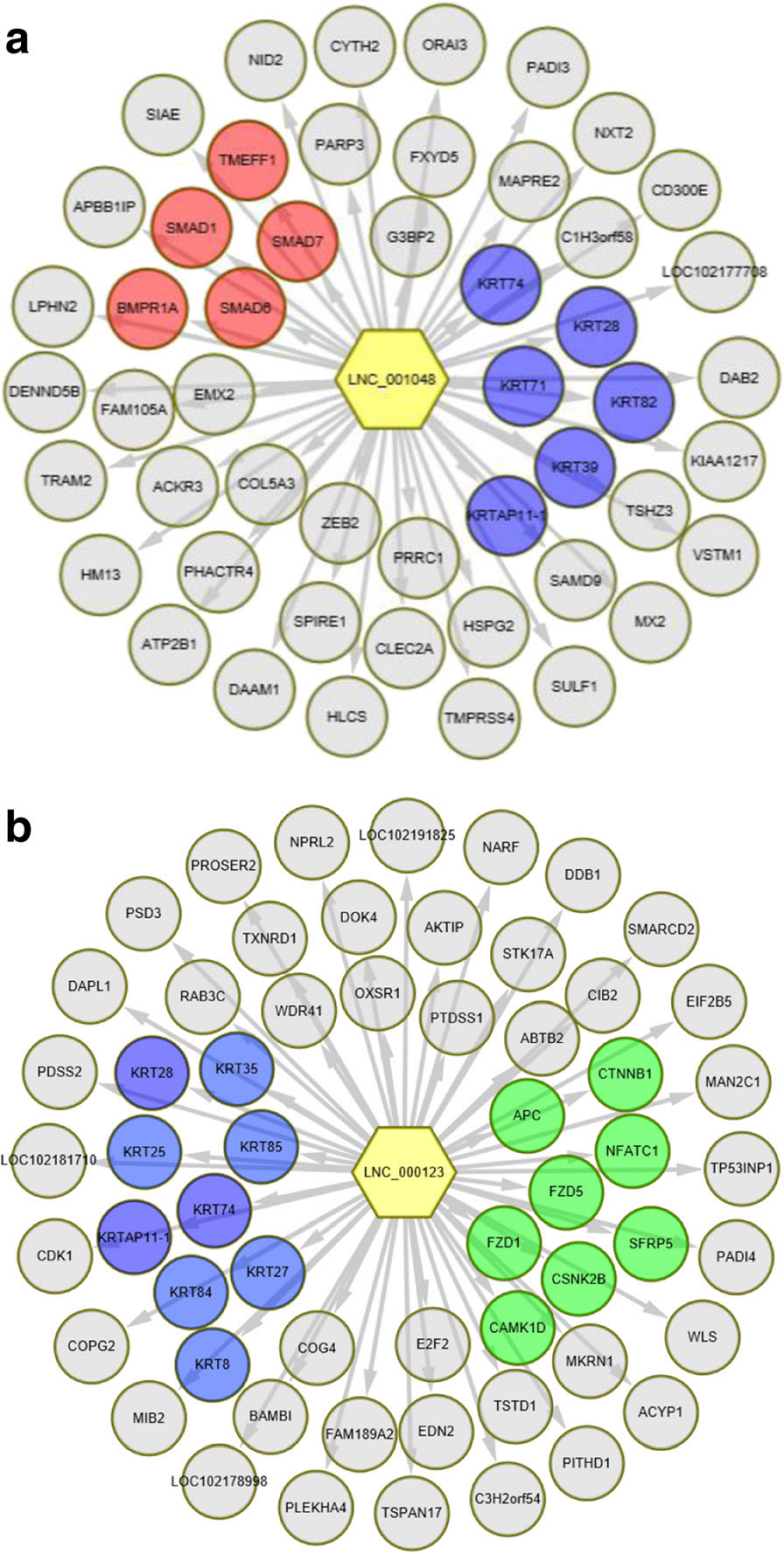
The potential targets of lnc_001048 and lnc_000123. **a** The potential targets of lnc_001048. **b** The potential targets of lnc_00123. One lncRNA may target multiple targets, red color indicates BMP/TGFβ signals, green color indicates WNT signal and blue color indicate KRTs. Grey circles represent other potential targets beyond BMP and WNT signals or KRTs. DAVID Bioinformatics Resources 6.7 were used to generate the pathways. Arrows indicate effect direction

To investigate the function of lncRNAs, the potential targets of lncRNAs in cis and trans were predicted. For the cis action of lncRNAs, we searched for protein-coding genes 100 kb upstream and downstream of the lncRNAs (Additional file 10). Interestingly, we detected HF cycling related genes such as *WNT3A*, *HOXC13* and *MSX2* which were located near the LNC_000972, LNC_000503 and LNC_000881 loci respectively (Table 1), suggesting that HF cycling may be regulated by the action of lncRNAs on neighboring protein-coding genes. On the other hand, the trans role of 1108 lncRNAs in protein-coding genes was examined based on its expression correlation coefficient (Pearson correlation ≥0.95 or ≤ −0.95) (Additional file 11). As a result, we found that the genes related with HF biology might be targeted by a few lncRNAs (Table 1). Moreover, some lncRNAs, like lnc_000123, lnc_000188 and lnc_000203, target multiple KRTs and KAPs, which suggested their potential function on keratin regulation. Specifically, Lnc_000123 targeted *APC*, *CTNNB1*, *NFATC1* and *FZD1* (Fig. 5a), and lnc_001048 targeted *BMPR1A*, *SMAD1*, *SMAD6*, *SMAD7* and *TMEFF1* (Fig. 5b), which indicated that they might participate in the WNT or BMP/TGFβ pathways, respectively.

**Table 1:** LncRNAs and its potential target genes that are involved in hair follicle cycling

Protein-coding genes	lncRNA in cis	lncRNA in Trans
Wnt related		
wnt3		LNC_000211, LNC_000126, LNC_000701, LNC_000826
wnt3a	LNC_000972	
wnt10a		LNC_000157, LNC_000640
APC		LNC_000814, LNC_000856, LNC_000924, LNC_001044, LNC_000471, LNC_000638
ctnnb1		LNC_000123, LNC_000194, LNC_000196, LNC_000217, LNC_000542, LNC_000693, LNC_00924, LNC_001012, LNC_001024
Tcf3		LNC_000519, LNC_000643
Tcf4		LNC_000662, LNC_000728
DKK3		LNC_000945, LNC_000971
BMP/TGFβ related		
TGFB1		LNC_000294
TGFBR1	LNC_001012	
BMPR1a		LNC_001050
FST		LNC_000074
BMP4		LNC_000038, LNC_000178, LNC_000808
Shh related		
DHH	LNC_000918	LNC_000544
Gli1		LNC_000633
Gli3	LNC_000842	LNC_000222, LNC_001006
Notch related		
notch2		LNC_000577, LNC_000178
RBPJ		LNC_001052
Hey1		LNC_000142
Hey2	LNC_001057	
DLK2		LNC_000820, LNC_000834
FGF/EGF related		
FGF10		LNC_000037
FGF18		LNC_000689
FGFR2		LNC_000176, LNC_000445, LNC_000999, LNC_000662, LNC_000914
TGFa		LNC_000885
Transcription factors		
Runx1		LNC_000222, LNC_000577, LNC_000825
sox9	LNC_000446	LNC_000648, LNC_000727
LHX2	LNC_000185	LNC_000398,LNC_000422
Dlx3		LNC_000708
Msx2	LNC_000503	LNC_000287, LNC_000385, LNC_001041
Gata3		LNC_000181
Tp63		LNC_000409, LNC_000516, LNC_001040
Foxn1		LNC_000164, LNC_000205, LNC_000316, LNC_000694
Hoxc13	LNC_000881	LNC_000269, LNC_000168

To further ascertain lncRNA-protein coding gene pairs belonging to both co-localization (cis action) and expression correlation (trans action) relationships, detailed examination was conducted. Eighty-four lncRNA-protein coding gene pairs that fulfilled these criteria were identified (Additional file 12). This finding suggested that lncRNAs act on their neighboring protein-coding genes to regulate gene expression.

### lncRNA as the precursor of miRNA and competing endogenous RNA (ceRNA)

LncRNAs can be small RNA precursors and can also negatively regulate miRNA maturation [51]. When the lncRNA and miRNA sequencing data was combined, all the lncRNAs overlapped precursors of miRNAs from genome-wide miRNA predictions. We found 12 lncRNAs that were possible precursors of 11 miRNAs (Additional file 13).

**Fig. 6:**
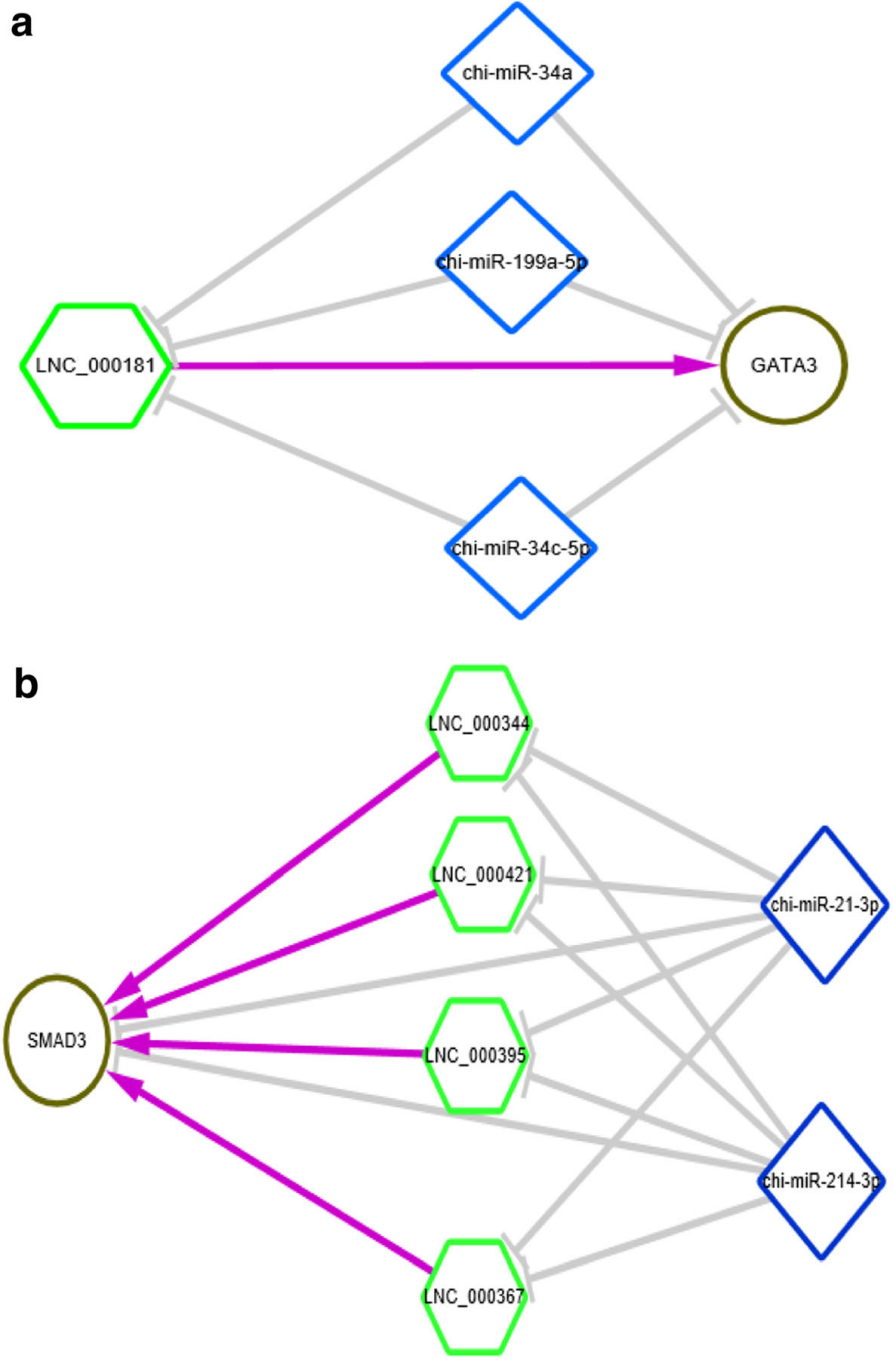
LncRNA function as ceRNA. **a** lnc_000181 serve as ceRNA to upregulate GATA3, (**b**) lnc_000344, lnc_000367, lnc_000395 and lnc_000421 may serve as ceRNAs to upregulate SMAD3. Hexagon indicates differently expressed lncRNA between anagen and telogen. Rhombus indicates miRNA involved in HF cycling and development. Circle indicates differently expressed gene between anagen and telogen. LncRNA-mRNA interactions were obtained based on its expression correlation coefficient (Pearson correlation ≥0.95). LncRNA-miRNA interactions were predicted by miRanda, mRNAs targeted by miRNAs were predicted by miRanda and targetscan. Gray line indicates negative regulation, purple line indicates positive regulation

It has been shown that lncRNAs function as ceRNAs by binding to and sequestering specific miRNAs in both plants and animals [27], and some miRNAs have been reported to regulate HF development and cycling. We predicted lncRNA that might act as ceRNAs using strict rules (see Methods). It showed that some lncRNAs could bind to specific miRNAs that related to HF cycling to protect the target mRNAs from repression and thus play an important role in HF biology as ceRNAs, such as miR-34a-lnc_000181-*GATA3* and miR-214-3p-lnc_000344-*SMAD3* ceRNA networks (Fig. 6). Lnc_000181 and lnc_000344 serve as ceRNAs to upregulate *GATA3* and *SMAD3*, respectively. The entire results were shown in Additional file 14.

## Discussion

Hair is a characteristic feature of mammals and performs a variety of roles, such as thermal insulation, physical protection, camouflage, social interaction and sensory perception. HF consists of multiple different cell populations of neural crest, ectodermal or mesodermal origin, which are distinct in their location, function and gene and protein expression characteristics [52, 53]. Additionally, the HF is a stem cell-rich and uniquely dynamic mini-organ that under-goes continuous cycling throughout adult life, so it could serve as a perfect model for systems biology research and organ regeneration [54].

### Regulation of HF cycling

The HF cycle has been traditionally subdivided into three stages: anagen (growth), catagen (cessation of growth) and telogen (resting), during which a number of morphological changes take place [55]. This cycle is regulated by complex and intricate interactions between the epithelial cells of the follicle and mesenchymal cells of the dermal papilla (DP) [5] and requires the spatiotemporal integration of multiple stimulatory and inhibitory signals [7, 56, 57], of which WNT and BMP/TGFβ are critical pathways [8, 58–60]. Corresponding with that, these pathways were also found in our current study. Note that previous studies mainly focused on human or mouse, however, cashmere growth is a seasonal phenomenon influenced by photoperiod, which is different to human or mouse. Studies have shown that melatonin plays a critical role in cashmere cycling and development under the control of photoperiod [61]. Melatonin may work through interacting with PRLR and DIO2 [62–64]. These two genes were found to have significantly different expression between anagen and telogen in this current study.

### Regulation of keratins during HF development

The primary structural proteins of hair fibers are the hair keratins and the KAPs. During HF development, a notable feature is the orderly expression of the keratins and KAPs [53]. Corresponding with that, we found a set of genes belonging to keratin family members and KAPs, which were markedly up-regulated in anagen vs. telogen. Keratins are intermediate filament proteins that have essential functions in maintaining the structural integrity of the epidermis and its appendages. Cell-specific keratin expression and organization impact on cell growth, migration and invasion. Their expression is under strict control to produce keratins that are optimally adapted to their environment [50, 65]. Transcription factors and other regulatory genes like *LEF1*, *SP1*, *HOXC13*, *FOXN1*, *DSG4*, *AP1* and *AP2* are critical factors that could regulate keratins [66–69]. Consistent with this, significant differences were observed between the expression of these genes in anagen and telogen, which verified their interactions in HF.

### MiRNAs and lncRNAs play an important role in HF cycling

Beyond coding genes, ncRNAs play an indispensable role in HF biology. Previous studies have identified a few miRNAs and revealed their function in HF morphogenesis and development, such as miR-214, miR-21, miR-24 and miR-196a [22–24]. However, these studies only focused on the HF biology of humans and mice, little was known about cashmere goat HF biology, specifically cashmere cycling. We found that miR-214 and miR-196a might also play an important role in HF cycling of cashmere goat. Other studies revealed that lncRNAs play important regulatory roles in gene expression and contribute to skin biology [25], whereas there had been no related reports on cashmere cycling. In this study, we have identified lncRNAs in different HF stages of cashmere. We identified 1108 lncRNAs in total, some of which were adjacent or co-expressed with HF development related genes, which indicated their possible functions on HF cycling. These will greatly enrich the lncRNA database in Cashmere goat.

### LncRNA could function as ceRNA during HF cycling

Nowadays, the influence of lncRNAs upon miRNA function is also rapidly emerging. In some cases, lncRNA stability is reduced through the interaction of specific miRNAs. In other cases, lncRNAs can act as miRNA decoys, with the sequestration of miRNA favoring expression of repressed target mRNAs. Other lncRNAs depress gene expression by competing with miRNAs for interaction with shared target mRNAs. Finally, some lncRNAs can produce miRNAs, leading to repression of target mRNAs [27, 28, 70]. These miRNA–lncRNA interactions are central to mammalian physiological and pathological processes [71, 72].

In current study, we constructed the ceRNA networks jointed by lncRNAs, miRNAs and mRNAs based on lncRNA and miRNA sequencing data. To enhance data reliability, defined rules were set to screen candidate ceRNAs. Our analysis has suggested that lncRNAs harbor potential miRNA recognition elements and participate in a complex ceRNA network. The network brings to light an unknown miRNA regulatory network in cashmere cycling. It also suggests that lncRNAs may play crucial roles in cashmere cycling and maintenance.

## Conclusions

In conclusion, in this study, we present the first data on the lncRNA of cashmere cycling. Combined with the miRNA sequence data, the ceRNA networks were constructed, which expands our knowledge about lncRNA and miRNA biology and contributes to the annotation of the goat genome as well.

## Additional files

Additional file 1: Primers used in quantitative PCR analysis. (XLSX 9 kb)

Additional file 2: Novel lncRNA information. (XLSX 85 kb)

Additional file 3: The sequence of lncRNA. (TXT 2937 kb)

Additional file 4: Annotated mature miRNAs and precursors. (XLSX 17 kb)

Additional file 5: Novel mature miRNA sequence and precursor sequence. (TXT 14 kb)

Additional file 6: Differentially expressed genes between anagen and telogen. (XLSX 229 kb)

Additional file 7: Differentially expressed lncRNA between anagen and telogen. (XLSX 26 kb)

Additional file 8: Differentially expressed KRTS and KAPs. (XLSX 14 kb)

Additional file 9: Differentially expressed miRNAs between anagen and telogen. (XLSX 15 kb)

Additional file 10: The protein-coding genes within 100 k upstream and downstream of an lncRNA. (XLSX 310 kb)

Additional file 11: Pearson correlations between protein-coding genes and lncRNAs. (XLSX 1804 kb)

Additional file 12: LncRNA-protein coding gene pairs with both colocalization and correlation relationships. (XLSX 12 kb)

Additional file 13: LncRNA as miRNA precursor. (XLSX 10 kb)

Additional file 14: Potential lncRNAs function as competing endogenous RNAs (ceRNAs) duing hair follicle cycling. (XLSX 17 kb)

## Abbreviations

DEG: Differentially expressed gene
HF: Hair follicle
KAP: Keratin-associated protein
KEGG: Kyoto encyclopedia of genes and genomes
KRT: Keratin family member encoding gene
lincRNA: Long intergenic non-coding RNA
lncRNA: Long non-coding RNA
miRNA: microRNA
ncRNA: Non-coding RNA
PAGE: Polyacrylamide gel electrophoresis
qRT-PCR: Quantitative real-time PCR
